# Enhancing Retention in Digit Rehabilitation: Implant-Assisted Silicone Finger Prosthesis for an Amputated Thumb

**DOI:** 10.7759/cureus.67596

**Published:** 2024-08-23

**Authors:** Kyathi K Jathan, Shivsagar Tewary, Nilesh R Mishra, Vijaysinh H Patil, Pronob Sanyal

**Affiliations:** 1 Prosthodontics and Crown and Bridge, School of Dental Sciences, Krishna Vishwa Vidyapeeth, Karad, IND; 2 Oral and Maxillofacial Surgery, School of Dental Sciences, Krishna Vishwa Vidyapeeth, Karad, IND; 3 Orthopaedic Surgery, Krishna Institute of Medical Sciences, Krishna Vishwa Vidyapeeth, Karad, IND

**Keywords:** maxillofacial prosthesis, thumb, custom abutment, osseointegrated prosthesis, implant-retained, finger prosthesis

## Abstract

This case report details the prosthetic replacement of a thumb lost due to traumatic amputation. The primary goal of this replacement was to restore the thumb's functionality, enabling the patient to resume daily activities such as writing and holding objects. A silicone prosthesis, anchored by an osseointegrated implant in the metacarpal bone, was used for this purpose. In this instance, a young female's thumb stump was functionally rehabilitated with the assistance of bone-anchored implants and room-temperature-vulcanizing silicones. At the follow-up appointment, no complications were observed, and the prosthesis remained in excellent condition, requiring no additional intervention. Utilizing bone-anchored implants to enhance retention in short stumps post-amputation proves to be one of the most effective methods to restore function and improve the daily lives of such patients.

## Introduction

Both the function and appearance of the hand are essential attributes. Hands contribute to aesthetics, enhancing the beauty of gestures and the elegance of movements [1]. Various conditions, including congenital abnormalities and diseases, can affect hands, but trauma is the most significant cause of functional impairment. Traumatic finger amputations severely impact hand function, leading to substantial impairment [2]. While many severely injured or amputated digits can now be saved through microsurgical replantation, reconstruction is not always possible or successful in some patients. For this group, prostheses can be a viable option, providing not only functional benefits but also substantial psychological support for the loss of confidence and self-esteem that comes with the loss of a finger [2].

From a functional perspective, the thumb accounts for at least 50% of the hand's utility [3], and hence its replacement holds great significance in terms of re-establishing the lost grips and grasps that make the hand functional. The advancement of osseointegrated digital prostheses has significantly improved prosthetic design and function [4]. This technique provides a viable alternative for patients with short stumps that cannot accommodate standard digit prostheses. The prosthesis is securely attached via an osseointegrated implant inserted into the intramedullary canal of the remaining bone of the amputated digit [4]. In this case report, this concept of using skin-penetrating implants to anchor external prostheses is extended to digit prostheses with the main aim of improving retention and thereby making the prosthesis functional.

## Case presentation

**Figure 1:**
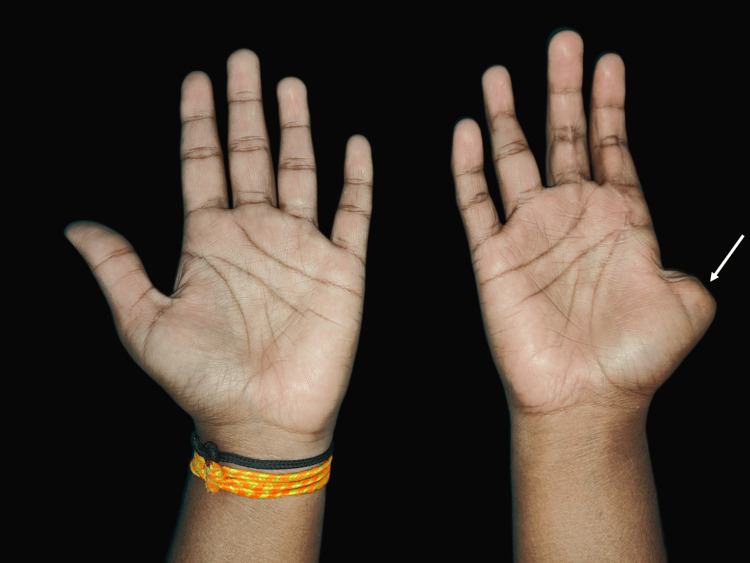
Palmar view of both hands showing missing thumb in the right hand (arrow)

A 22-year-old female patient reported to the Department of Prosthodontics and Crown & Bridge, School of Dental Sciences, Krishna Vishwa Vidyapeeth, Karad with the complaint of missing thumb of right hand for one year (Figure 1).

**Figure 2:**
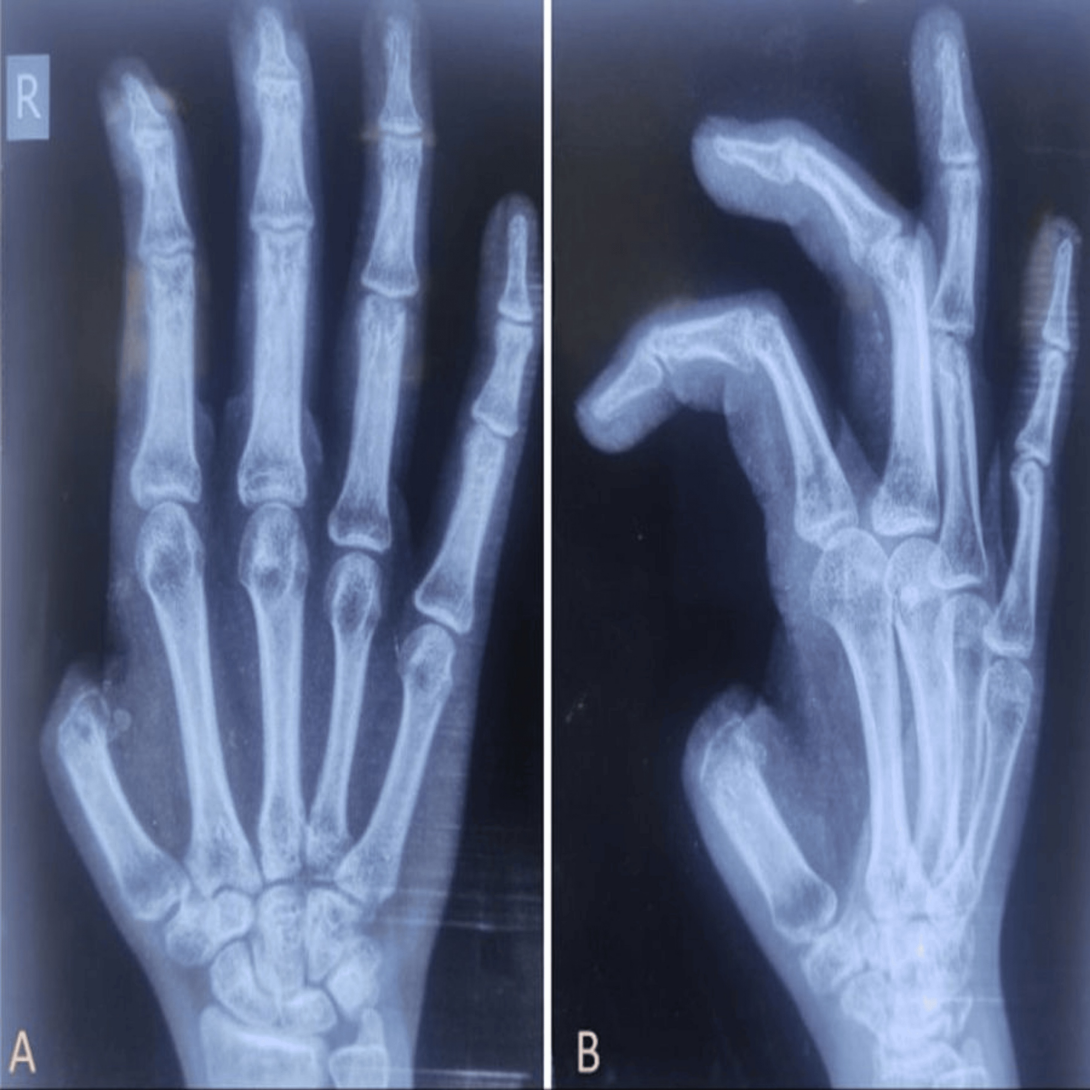
(A) Anteroposterior- and (B) oblique-view radiographs of the affected hand

The patient's medical history revealed that her right thumb had been amputated a year ago after being crushed under a thresher machine, for which she had undergone stump closure surgery. She was not distressed by the aesthetic disfigurement but was depressed due to her inability to perform certain tasks, especially writing. Physical examination and radiographs (anteroposterior and oblique views) (Figures 2A, 2B) of her right hand showed that the thumb had been amputated through the metacarpophalangeal (MCP) joint.

The rehabilitation was particularly challenging due to the location and level of the amputation. The remaining stump, approximately 1.2 cm in length, was insufficient for achieving adequate retention with a traditional finger prosthesis. Consequently, the decision was made to proceed with an implant-retained thumb prosthesis for functional restoration.

**Figure 3:**
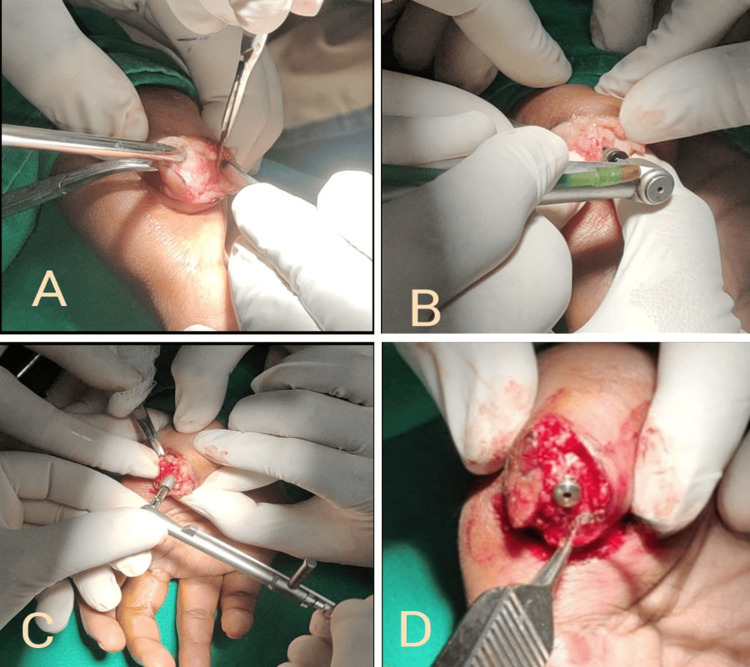
Surgical images A: Exposure of the metacarpal bone. B: Osteotomy site preparation. C: Implant placement with a torque wrench. D: Gingival former placed over the implant (arrow)

The patient was informed about the procedure and she provided her consent. Blood tests were conducted and all results were within the normal range, allowing for the implant placement to proceed. Under local anesthesia, an endosseous implant (Genesis Biocon, Noida, India) measuring 5 x 13 mm was inserted into the intramedullary canal of the metacarpal bone in the right thumb (Figures 3A-3C). A healing abutment was placed to ensure patency (Figure 3D).

**Figure 4:**
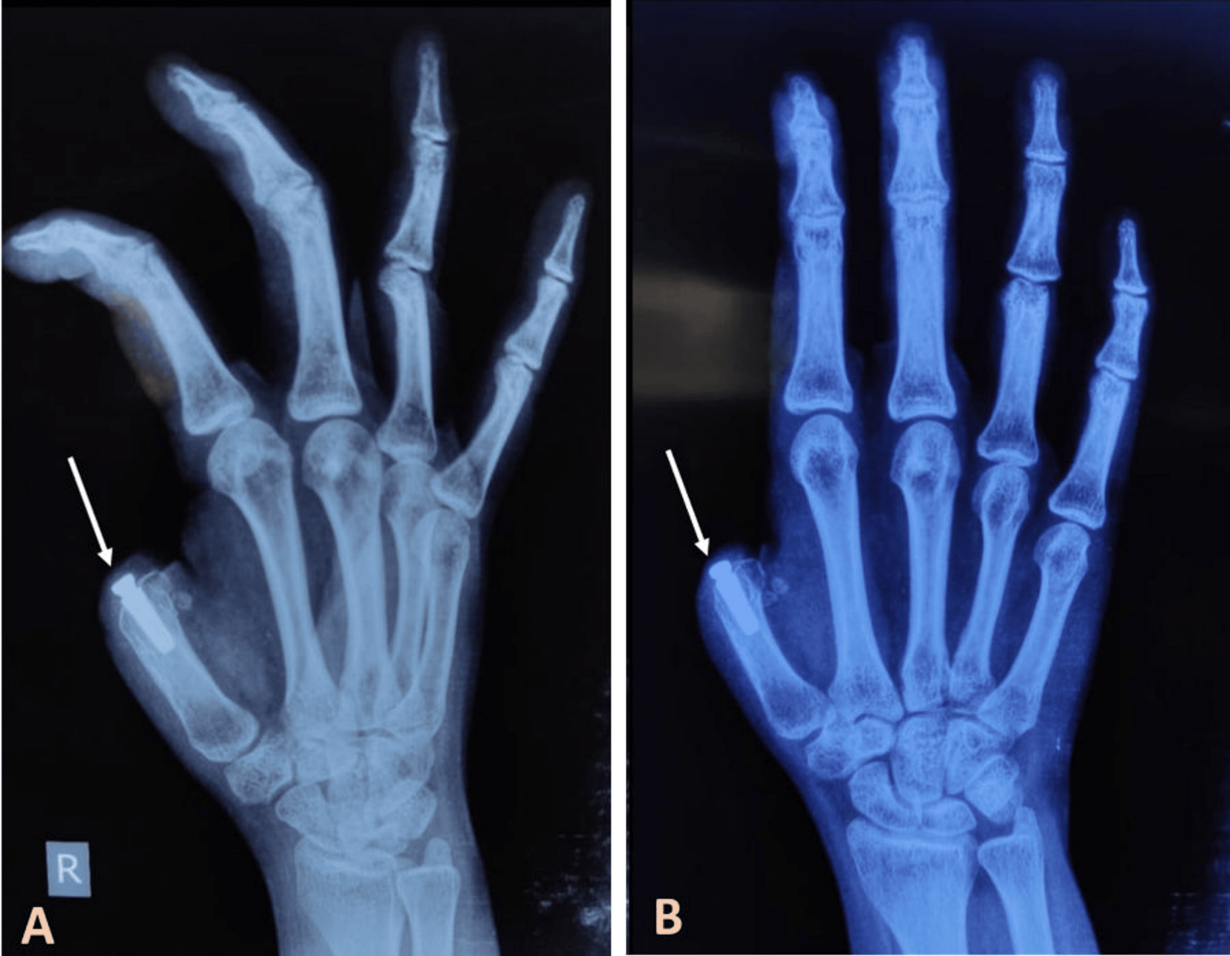
(A) Oblique- and (B) anteroposterior-view radiographs of the right thumb showing an osseointegrated implant (arrows)

Three months later, osseointegration was confirmed through another set of radiographs (anteroposterior and oblique views) (Figure 4).

**Figure 5:**
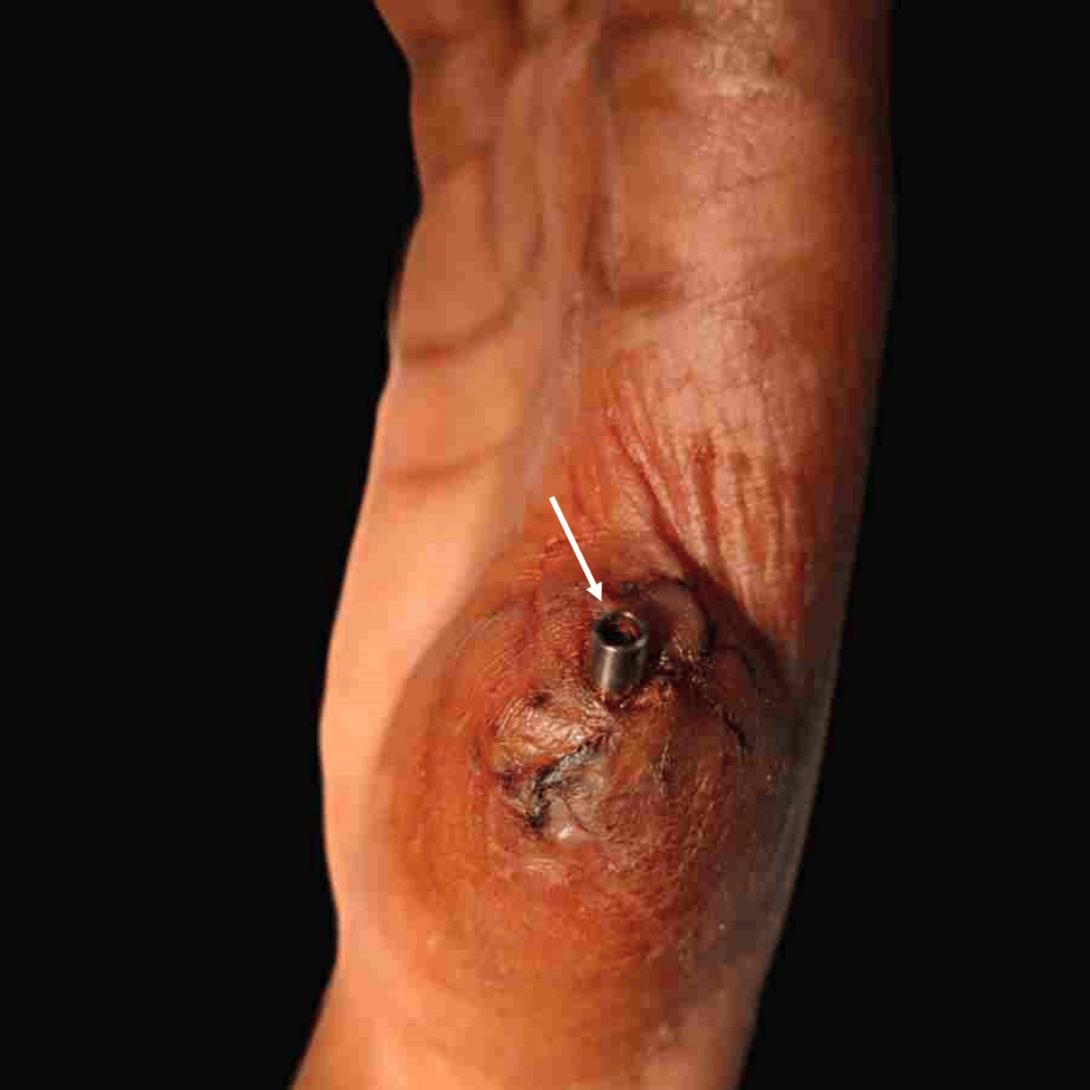
Straight abutment placed to maintain patency (arrow)

Subsequently, a second-stage surgery was performed to expose the implant, and a straight abutment (Genesis Biocon) was placed to maintain patency and establish a proper emergence profile (Figure 5).

**Figure 6:**
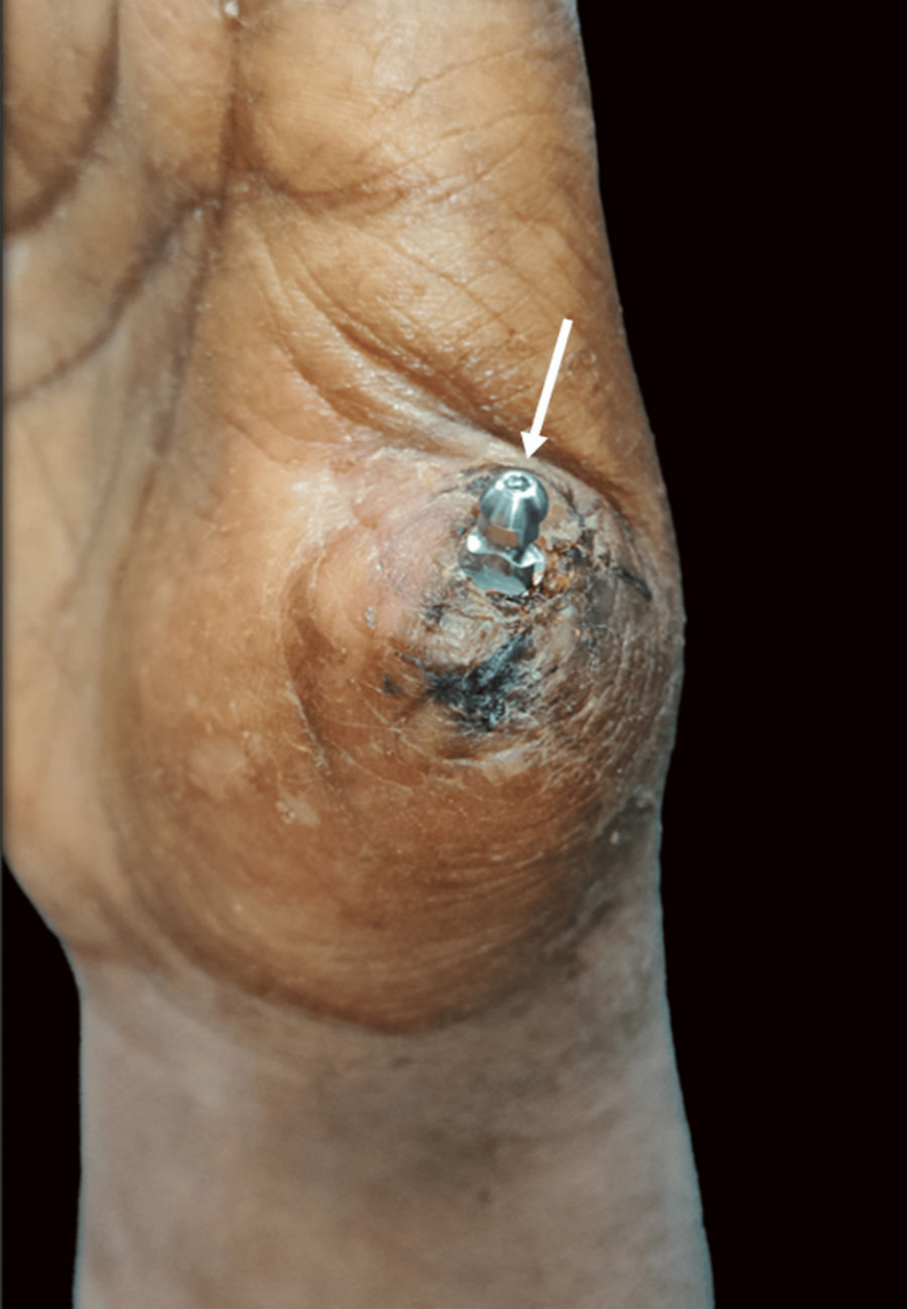
Closed tray impression coping placed for making an alginate impression of the stump (arrow)

After a brief period of three weeks, the prosthetic steps were initiated. The straight abutment was removed and the emergence profile was evaluated. A closed tray impression coping was placed (Figure 6).

**Figure 7:**
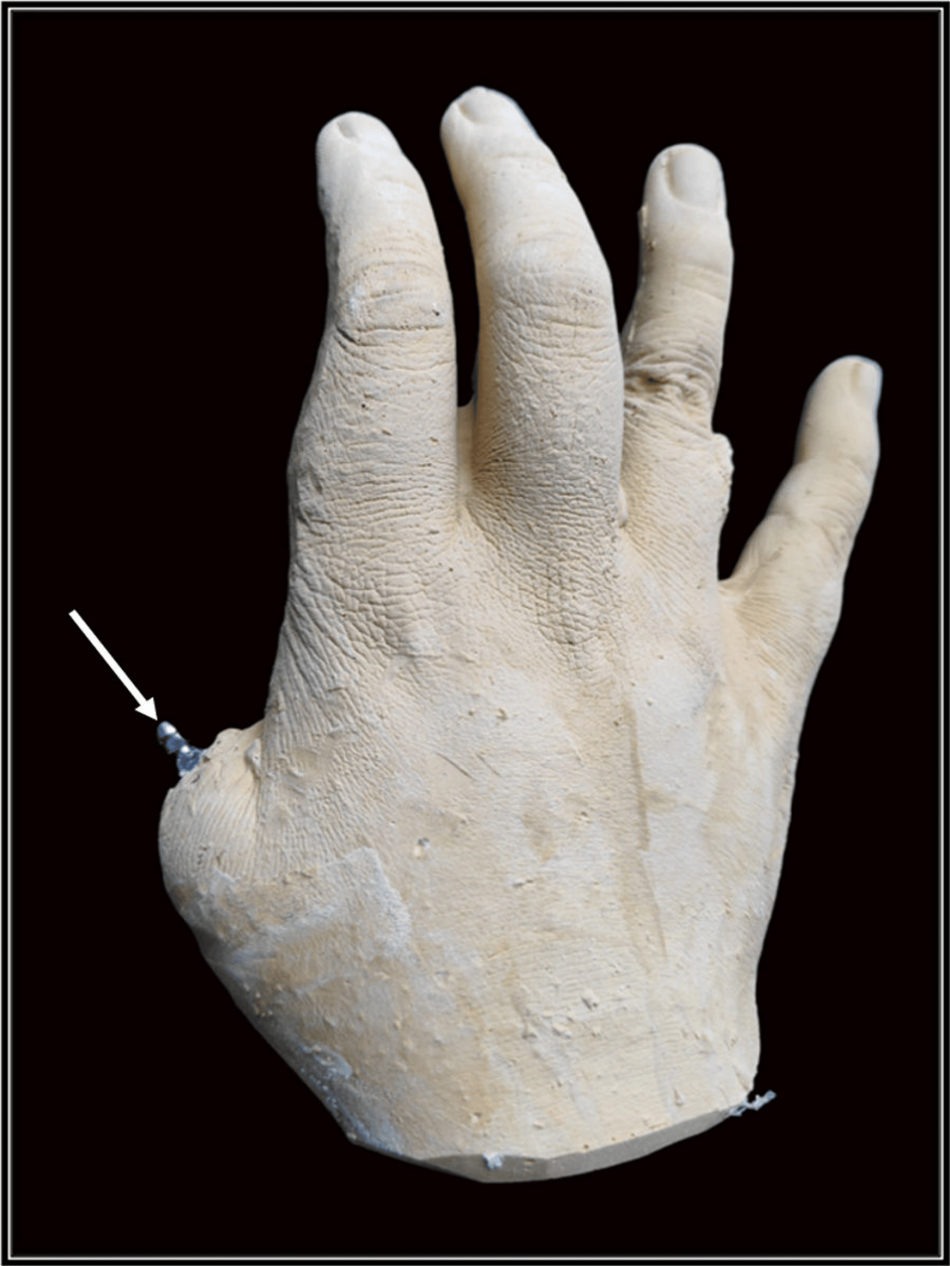
Secondary cast with impression coping (arrow)

This was followed by making an alginate impression (Septodont, Saint-Maur-des-Fossés, France). A cast with the impression coping was created using dental stone (Kalstone, Kalabhai Pvt. Ltd., Mumbai, India) (Figure 7).

**Figure 8:**
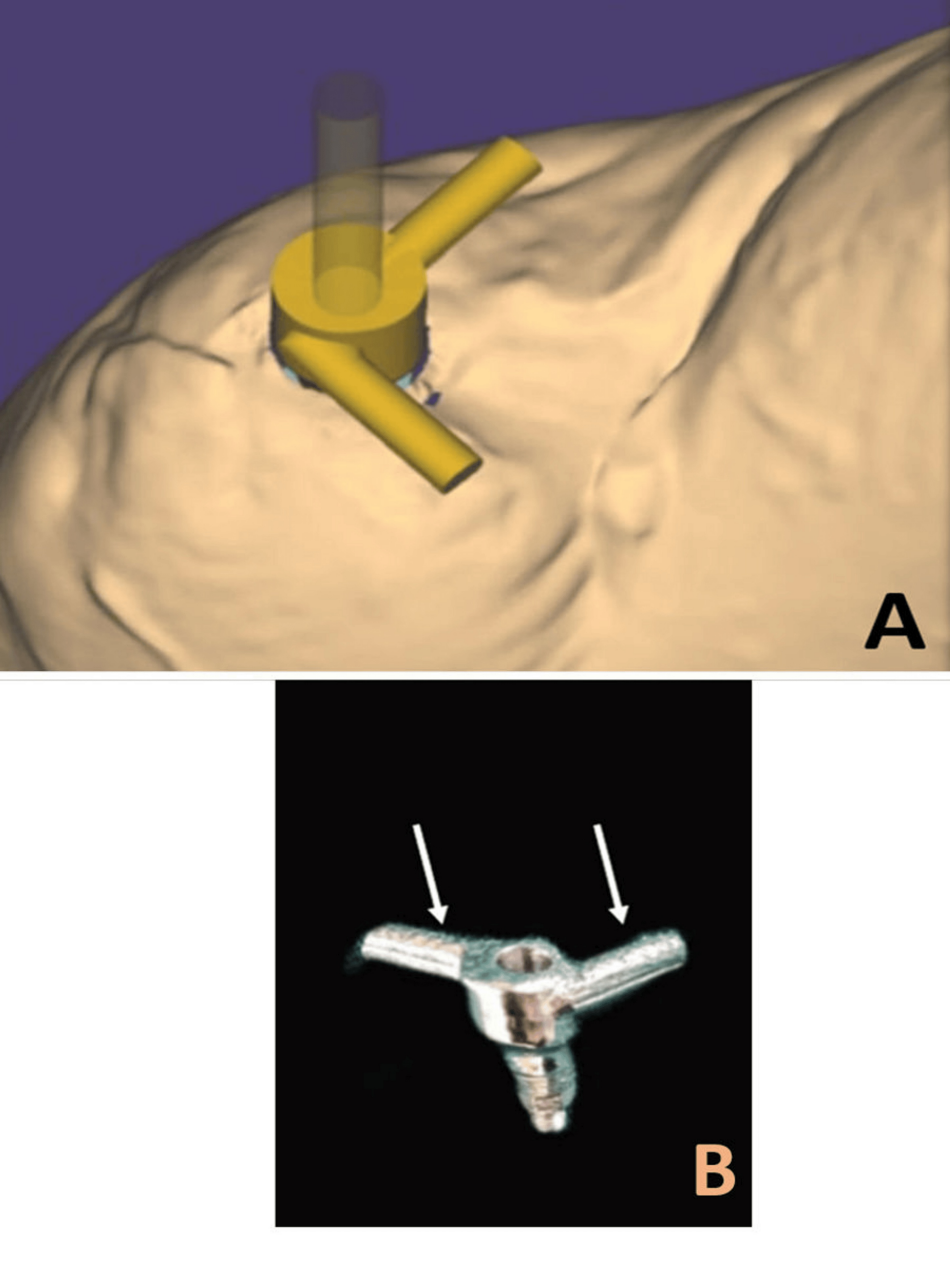
A: Computer-aided designing of the custom abutment. B: Direct metal laser-sintered custom abutment with anti-rotational bars (arrows)

This cast was scanned, and a custom abutment was designed with computer-aided design (Exocad GmbH, Darmstadt, Germany) (Figure 8A), featuring anti-rotation bars to prevent movement in any plane during use and to support the metal housings which would prevent dislodgement and improve retention along with an access hole that would allow the abutment to be screwed over the implant (Figure 8B). The abutment was milled using a direct metal laser-sintered type of additive manufactured with cobalt-chromium alloy.

**Figure 9:**
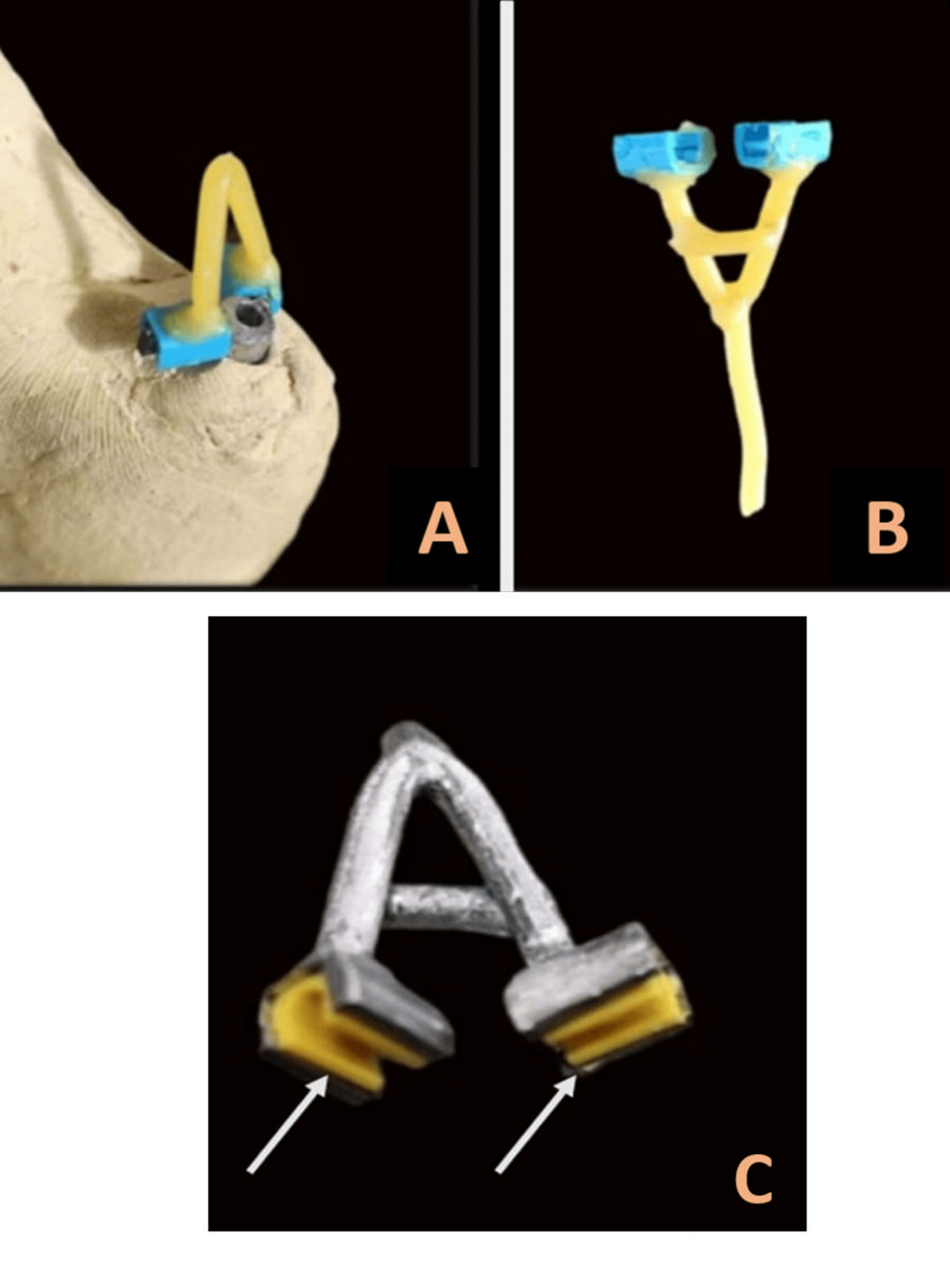
A: Plastic sleeves for the metal housings assembled along with the wax pattern of the retentive framework. B: Wax pattern of retentive element for silicone. C. Cast retentive framework along with retentive clips (arrows) in the metal housing

A retentive element with metal housings was designed on top of this milled abutment to secure the silicone prosthesis, preventing it from dislodging during placement and removal (Figure 9A). The retentive framework was cast in cobalt-chromium alloy and the retentive clips were picked up in the cast metal housings to form the retentive framework (Figure 9B).

**Figure 10:**
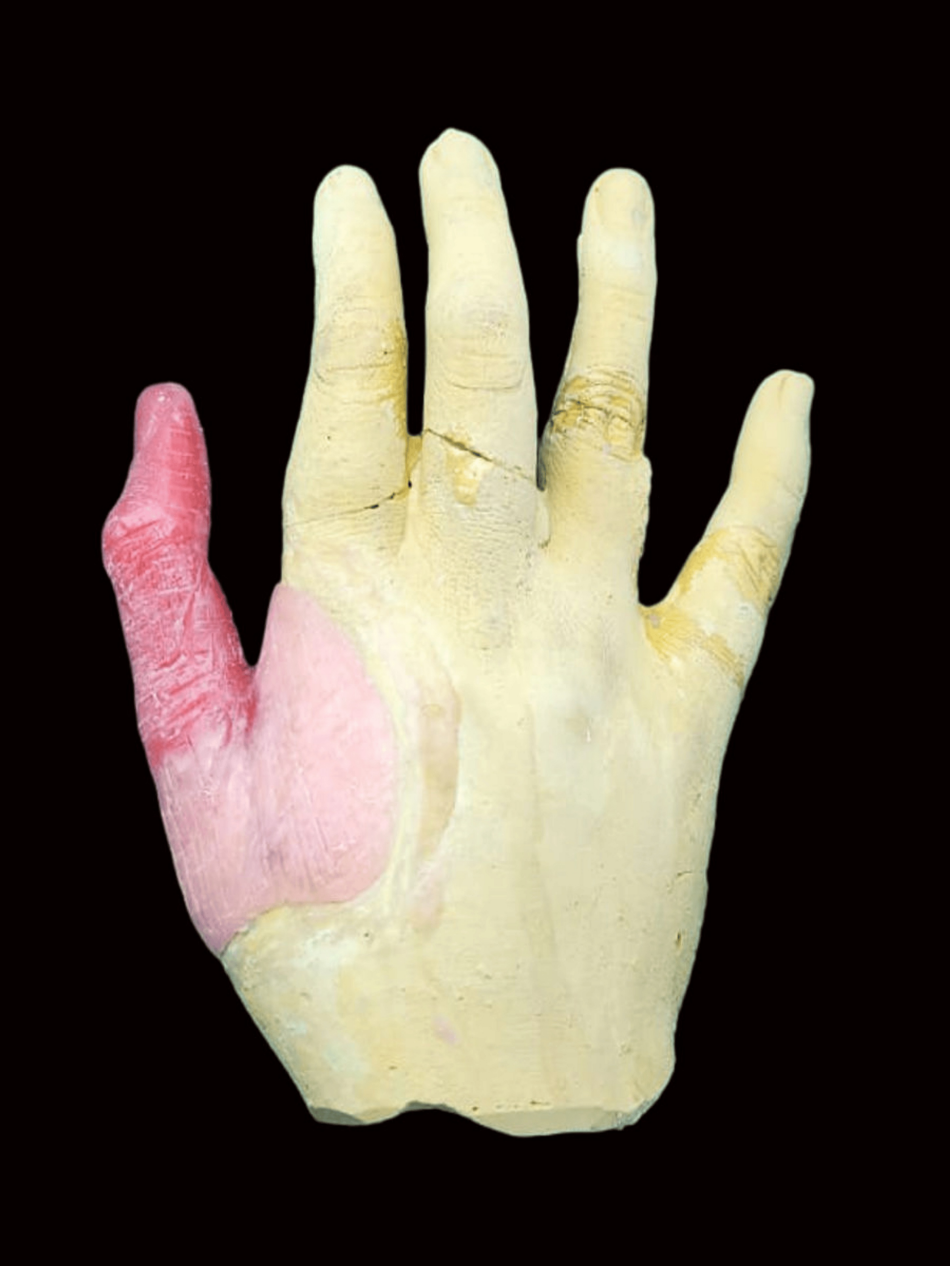
Wax pattern fabrication on the cast

The entire assembly was tested on the cast and then verified on the patient’s thumb. A wax pattern of the thumb was manually fabricated with a bend of approximately 20 degrees to improve precision and power grip which was then packed and dewaxed (Figure 10).

**Figure 11:**
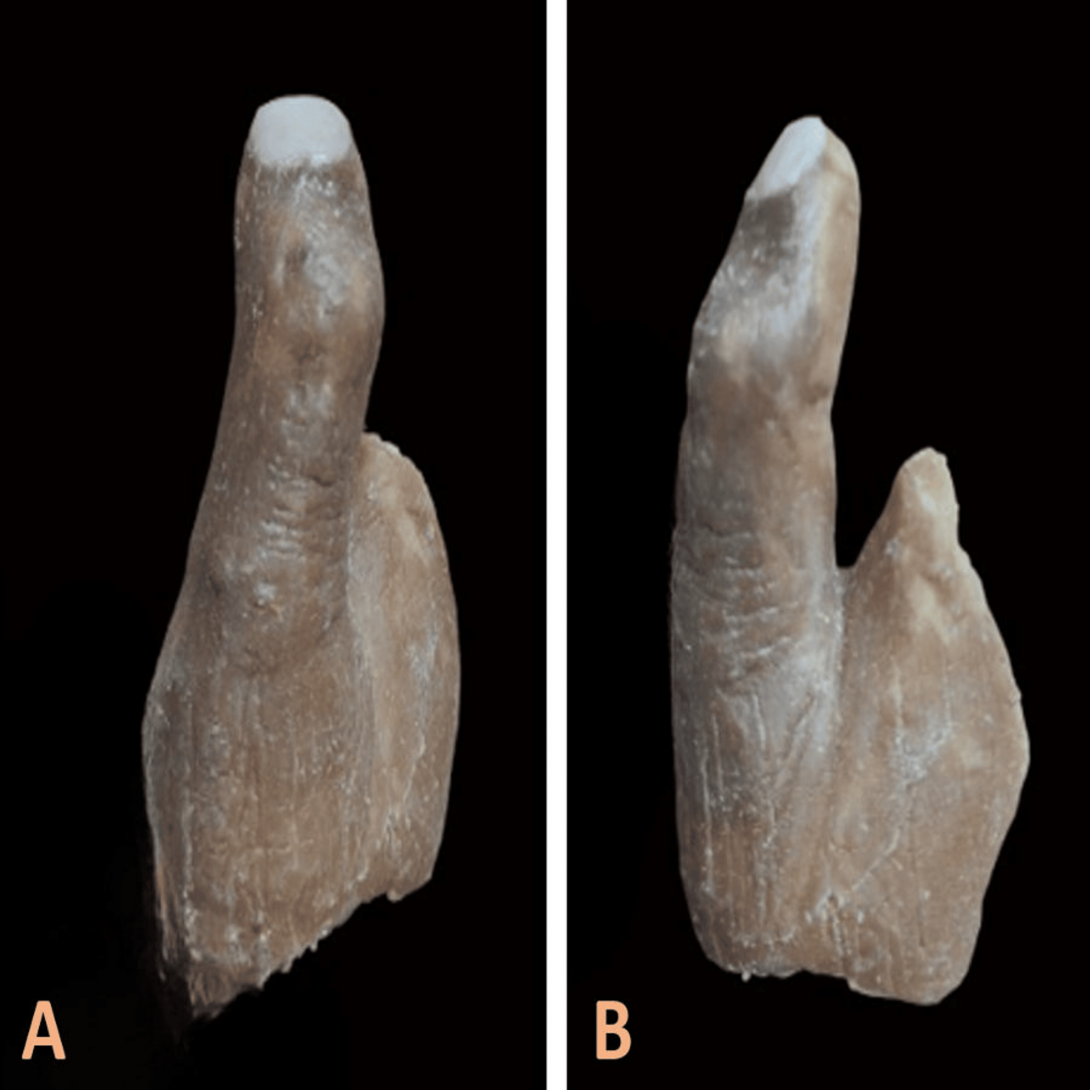
(A) Dorsal and (B) axial views of the retrieved finger prosthesis

Following this, room-temperature-vulcanizing silicone (Technovent Ltd., South Wales, UK) was packed into the mold after shade matching and allowed to cure at room temperature for 24 hours following which the prosthesis was retrieved (Figure 11).

**Figure 12:**
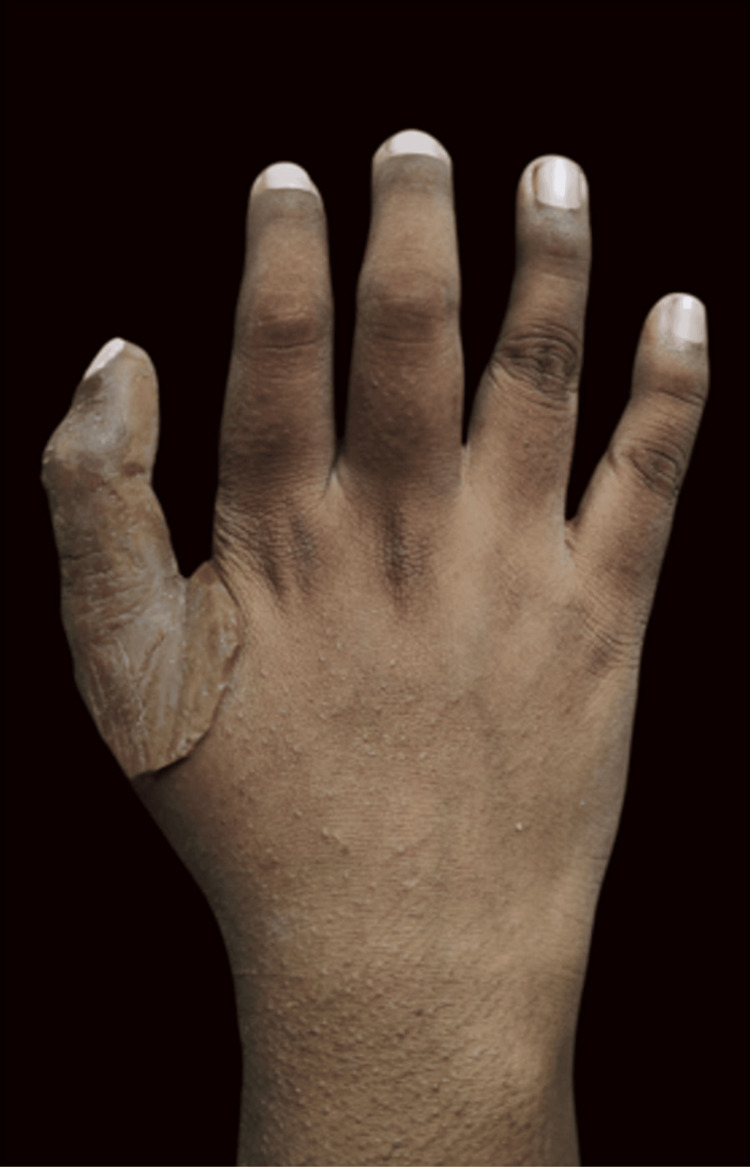
Implant-retained thumb prosthesis insertion

It then underwent final polishing and external staining; the retentive clips were housed at the base of the prosthesis and then fitted onto the patient’s thumb (Figure 12).

**Figure 13:**
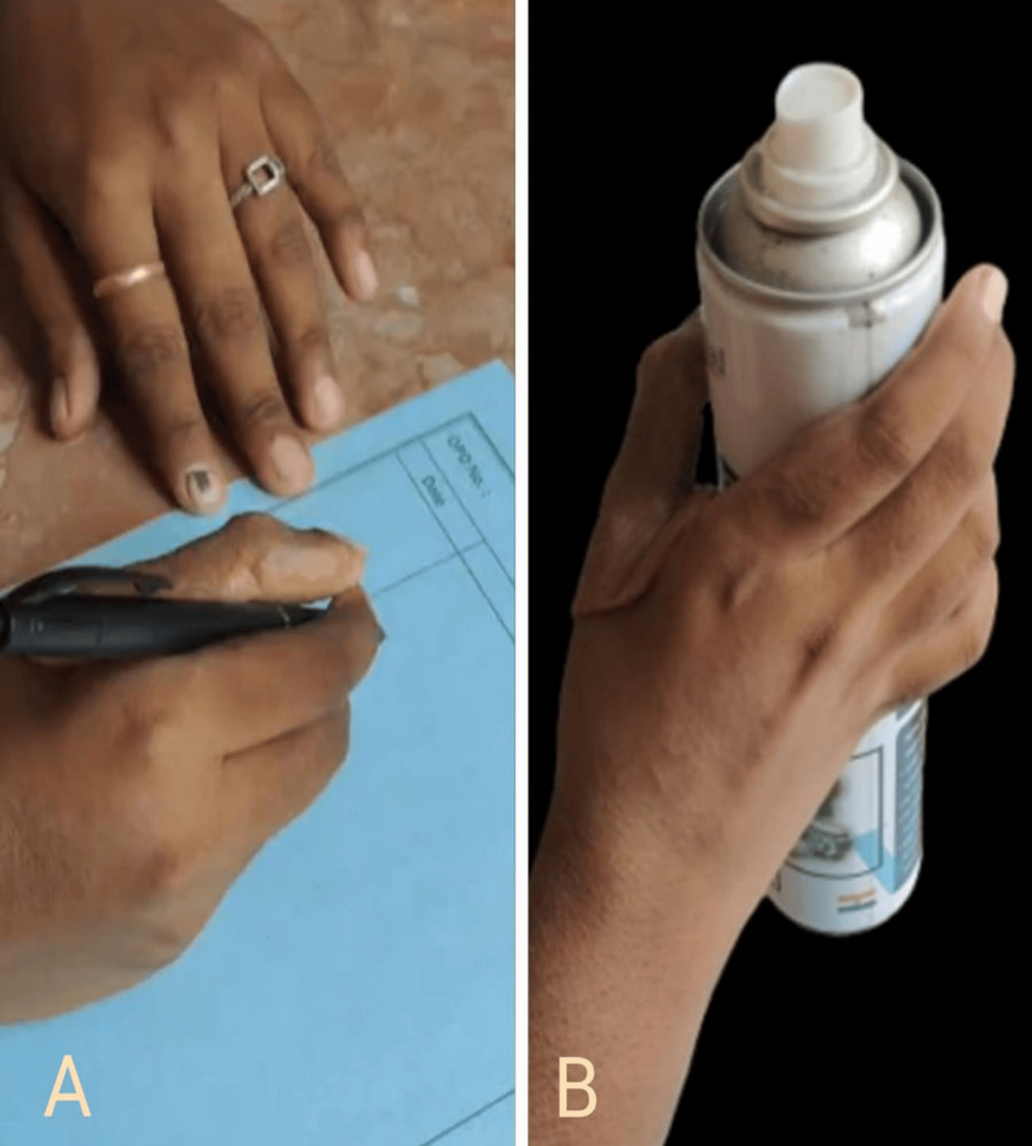
Functions restored with the help of the finger prosthesis - A: writing; B: grasping onto a bottle and lifting it

The patient was able to resume writing (Figure 13A) and could also hold and lift a bottle effectively (Figure 13B). The primary goals of her seeking treatment were successfully achieved, providing her with the functional replacement of the thumb.

The prosthesis was inserted and the patient was advised in-home care and prosthesis maintenance. Home care included mechanical debridement of the skin around the abutments with a soft toothbrush and irrigation with warm water and soap. The patient was kept on regular follow-ups, and it was observed that the skin around the attachments appeared healthy and retention of the prosthesis was satisfactory.

## Discussion

Patients who have undergone surgical removal of diseased tissues need prompt restoration of function and aesthetics to reduce psychological trauma. The traumatic amputation of the thumb presents a major deformity, causing significant functional and aesthetic difficulties [5]. The thumb and index finger play crucial roles in collateral and precision grip, the two most essential prehensile functions of the hand. Therefore, reconstructing the thumb and index finger is of utmost importance [6].

Modern prosthetic designs for digital prostheses offer excellent aesthetics and can be crafted to provide a stable point for light grasping. A review of the literature indicates that clinical reports on digital prostheses are relatively scarce [7]. Retention for finger prostheses can be achieved through various methods, including vacuum effect, rings, straps, or implants. For traditional finger prostheses, a stump height of at least 1.5 cm is required for adequate retention. If a stump height of 1.5 cm is not available, using implants is a reliable alternative for securing retention [8].

Retention is a crucial factor for the success of a finger prosthesis, and the use of osseointegrated prostheses has significantly enhanced this aspect. It is commonly observed that inserting an osseointegrated titanium implant into the intramedullary canal of the residual bone in an amputated stump provides a secure attachment for the prosthesis. Studies, including those by Lundborg et al. [8], have investigated the mechanism of osseoperception, revealing that tactile stimuli applied to the prosthesis activate the primary somatosensory cortex of the brain. This case report endorses previous findings, showing that osseointegrated finger prostheses significantly improve both aesthetics and function for patients with finger amputations [9]. 

The final thumb prosthesis was designed with a bend of approximately 20 degrees to improve both precision and power grip, enhancing the thumb's functionality in a way that a straight design would not achieve [10]. Patients who received osseointegrated finger prostheses experienced functional improvements without any discomfort, as reported in existing literature [11]. Bone-anchored implants can restore a wide range of motion and grip strength, not only improving the aesthetic appearance but also significantly enhancing the functional capabilities of individuals, thereby enabling them to perform daily tasks with greater ease and confidence [12]. The results observed in this case report corroborate the documented benefits of such treatments and demonstrate that it is an effective treatment modality with good clinical success.

## Conclusions

Utilizing implant-retained silicone prostheses for reconstructing amputated fingers offers valuable therapeutic benefits, encompassing aesthetics, functionality, and psychological well-being for patients. Through bone-anchored implants, a broad spectrum of motion and grip strength can be restored, thereby not only enhancing appearance but also significantly improving individuals' functional capacities. Thus, it can be aptly stated that "Hands without fingers are as devoid of function and utility as a mouth without teeth".
